# Prevalence of Low Back Pain and Associated Factors in Older Adults: Amazonia Brazilian Community Study

**DOI:** 10.3390/healthcare9050539

**Published:** 2021-05-05

**Authors:** Ingred Merllin Batista de Souza, Lilian Regiani Merini, Luiz Armando Vidal Ramos, Anice de Campos Pássaro, João Italo Dias França, Amélia Pasqual Marques

**Affiliations:** 1 Department of Physical Therapy, Speech-Language Pathology, and Audiology and Occupational Therapy, Faculty of Medicine at University of São Paulo, Cipotânea Street, Vila Universitária, 51, São Paulo 05360-000, SP, Brazil; luiz.armando@usp.br (L.A.V.R.); anicepassaro@usp.br (A.d.C.P.); pasqual@usp.br (A.P.M.); 2 Department of Faculty of Physical Education and Physical Therapy at Federal University of Amazonas, Manaus, Jauary Marinho Avenue, Coroado, Manaus 69067-005, AM, Brazil; merinililian@hotmail.com; 3 Dante Pazzanese of Cardiolgy Institute, Avenue Dr Dante Pazzanese, 500, Vila Mariana 69077-000, SP, Brazil; jitalo@outlook.com

**Keywords:** prevalence, low back pain, older adults, pain, functional disability

## Abstract

To investigate the prevalence of low back pain (LBP) and associated factors in the older adult Amazonia Brazilian community, a cross-sectional study was conducted to evaluate 700 participants that were ≥60 years old. Pain intensity and functional disability were assessed using the Numerical Pain Scale and the Roland Morris Questionnaire, respectively, and their sociodemographic, clinical, and behavior variables were collected, i.e., age, sex, education level, socioeconomic level, anthropometric measurements, physical activity, health perception, and emotional state. The punctual prevalence rates of LBP were 42.4% (95% CI: 38.2–46.6%), and for the last 365 days, these prevalence rates were 93.7% (95% CI: 91.3–95.6%), the mean pain and functional disability scores were 6.17 ± 2.13 and 11.30 ± 6.07, and the moderate-to-severe disability was 39.7%. Pain and functional disability were associated with sex, chronic diseases, body mass index (BMI), physical activity level, health perception, and emotional level. In conclusion, the prevalence of LBP was high (for both punctual and the last 365 days), but the variables associated with being female, fewer years of schooling, sedentary behavior, diseases related to diet and the cardiovascular system, and impaired emotional levels had a higher level LBP, even though they considered themselves in good health. These findings can aid with coordinated efforts from government and health professionals to help manage and promote the prevention of LBP by considering the older adult population’s needs in the state of Amazonas.

## 1. Introduction

Low back pain (LBP) is the leading global cause of years lost due to disability and its burden is growing with the aging of the population [1]. LBP is an extremely common symptom that is experienced by people of all ages [2,3,4]. In 2015, the one-time global prevalence of activity-limiting LBP was 7.3%, which implies that 540 million people were affected by it [1]. LBP is defined by the location of the pain, typically between the lower margins of the ribs and the gluteal folds [2]. It is commonly accompanied by pain in one or both legs, and some people with LBP have associated neurological symptoms in the lower limbs [5]. The symptoms are often related to aging [6,7], and although it is identified as a common health problem, its prevalence is little known in the older adult population [8,9].

A systematic review [10], with data from the international literature on the prevalence of LBP in the older adult population (in developing or developed countries), indicates a high prevalence of LBP among older people, ranging from 21.7 to 75%. In the first nationwide meta-analysis that investigated the prevalence of LBP in older people in Brazil, Leopoldino et al. [11] provided moderate-quality evidence that the punctual prevalence of LBP in older Brazilians was 25.0% (95% CI: 18.0–32.0%). From a national perspective, this finding supports the notion that LBP is one of the most relevant health conditions in old age. Due to its impact on disability, the older adult population experience greater dependence, vulnerability, and lower quality of life [12].

In addition, knowing the sociodemographic and behavioral profiles, work habits, and general health factors associated with LBP in this group is essential for the development of public policies that are aimed at controlling the problem, which would be based on preventive measures and/or therapeutic interventions [13]. Therefore, this study aimed to investigate the prevalence of LBP and associated factors in the older adult population in an Amazonia Brazilian community.

## 2. Materials and Methods

### 2.1. Study Design

This cross-sectional study followed the Strengthening the Reporting of Observational Studies in Epidemiology (STROBE) [14] guidelines.

### 2.2. Study Population

The inclusion criteria were older people that were ≥60 years old, both sexes, living in the urban area of Manaus, Amazonas. The participants were asked, “Did you feel pain in the lumbar spine (lumbar region) in the past 3 months, regardless of time or duration?” A lumbar region image was presented along with verbal questioning to obtain more specific pain location information, and to allow for the questionnaires to be answered more independently. This study considered the nonspecific LBP definition according to Maher et al. [15]. The exclusion criteria were people who underwent a surgical procedure on the spine [16], used a wheelchair, used assistive walking devices, and had cognitive impairment. Interviews were carried out in places with a large flow of people of all age groups, and the participants were informed about all stages of the study and that their participation would be voluntary; they could leave the study at any time without causing any harm and with our responsibility of maintaining data confidentiality. The study was approved by the Research Ethics Committee of the Faculty of Medicine of the University of São Paulo, São Paulo, Brazil (protocol number: 189/16), and it is part of a multicenter study entitled “Low back pain prevalence in older Brazilian adults from different populations”.

### 2.3. Sample Size

In order to calculate the sample size, an estimate of LBP prevalence was used based on a systematic review, which assessed the worldwide prevalence of LBP. The parameters used were as follows: total older adult population in the city of Manaus, Amazonas = 108,100 [17], adjusted mean prevalence of LBP in the last month = 23.2% (*p* = 0.232) [3], accuracy of 4% (*p* = 0.04), 95% confidence interval (*z* = 1.96), and allowing for a sample loss of 20% due to refusal and incomplete questionnaires. The total sample size of the study was 513 individuals of the older adult population presented in each of the cities included in the multicenter study. In addition, it was not necessary to perform a finite correction of the population because the calculated sample was less than 5% of the older adult population [18].

### 2.4. Evaluation

#### 2.4.1. LBP Prevalence: Punctual and for the Last 365 Days

For this study, an episode of LBP was considered as any pain between the last rib and the bottom of the gluteal folds lasting more than 24 h, preceded by 30 days without pain [2]; an episode of nonspecific LBP, which was defined as LBP that was not attributable to a recognizable or previously known specific pathology, was also accepted [15]. Prevalence was measured in terms of punctual pain (at the time of the interview) and from the last 365 days (any occurrence in the last year). To enable the exact identification of the lumbar region, the interviewees received an illustrative figure of the human body that specified the lower back with dotted lines. The frequency, duration, and radiating pain were also investigated; we assessed whether the LBP was sufficient to limit daily activities for more than one day and whether there was radiating pain to the lower limbs.

#### 2.4.2. Sociodemographic and Clinical Variables

Sociodemographic characteristics (sex, age, marital status, and self-reported skin color), education level, occupational activity (that was performed for most of their lives), emotional level, health perception, self-reported diseases, alcohol consumption, and smoking status were evaluated. Anthropometric variables [19] (weight and height) were used to calculate the stratified body mass index (BMI).

The physical activity level was assessed using the International Physical Activity Questionnaire (IPAQ short version) [20], which classifies physical activity levels according to the frequency, duration, and intensity of the individual’s activity in their free time, travels, and domestic and occupational activities.

#### 2.4.3. Pain Assessment

Pain intensity was assessed using the Numerical Pain Scale (NPS), which is an 11-point scale ranging from 0 to 10, with zero points indicating the absence of pain and 10 points indicating unbearable pain. The individuals were asked about the presence of pain, specifically located in the lower back. The NPS is widely used in studies of this nature to subjectively quantify pain intensity [21].

#### 2.4.4. Functional Disability

Functional disability was assessed using the Roland Morris Questionnaire (RMQ), which is an instrument that was translated and adapted for the Brazilian population [22] and is widely used in research and clinical practice to assess the disability associated with LBP. It consists of 24 questions that are related to the normal activities of daily living. Participants were asked to identify the items in the questionnaire that they considered challenging to perform on that day because of LBP. The questionnaire score ranges from 0 to 24 points; higher scores indicate a more significant functional disability. Scores above 14 points correspond to severe functional disability [21].

#### 2.4.5. Statistical Analysis

Qualitative variables were presented as absolute and relative frequencies, and quantitative variables were presented as means and standard deviations or medians and quartile intervals, if indicated. Thereby, the absolute and relative frequencies of the sociodemographic, clinical, and behavioral variables of all participants were calculated, including the pain intensity (mean, median, and standard deviation), the level of functional disability (mean, median, and standard deviation), the prevalence of punctual LBP and for the last 365 days, along with the respective 95% confidence intervals (CIs).

Fisher’s exact test [23] was used to verify the associations between the qualitative variables. To check the relationship between the quantitative variables and the two groups, Student’s *t*-test or the Mann–Whitney test was used. Analysis of variance (assumption of normality) or the Kruskal–Wallis test (nonparametric) was used when there were more than two groups [24]. The prevalence ratio (PR) of unbearable pain and functional disability was calculated. The robust Poisson model [25,26] was used to calculate the adjusted PR, adjusting for age and sex [27], with explicative variables that have a *p*-value < 0.15. The data were analyzed using IBM Corporation., 2010 SPSS version 19 Armonk, NY and R Core version 3.5.1 (R Foundation for Statistical Computing, Vienna, Austria). The significance level adopted was 5%.

## 3. Results

**Figure 1:**
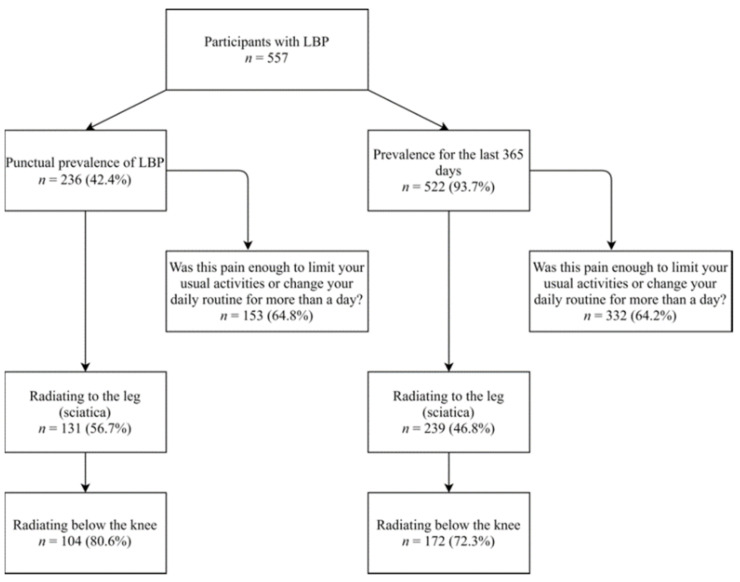
Flowchart of punctual prevalence and prevalence for the last 365 days.

We recruited 700 older adults and amongst them, 76.4% (95% CI: 76.4–82.5%) had LBP according to the study definition. Figure 1 shows a flowchart of the prevalence (punctual and for the last 365 days) of LBP in this study. The estimated mean for the punctual prevalence of LBP was 42.4% (95% CI: 38.2–46.6%), while the estimated mean for the prevalence in the last 365 days was 93.7% (95% CI: 91.3–95.6%). For those who reported having LBP at the time of the interview, the mean pain score was 6.17 points (SD: 2.13).

Regarding the mean for functional disability, the score was 11.3 points (SD: 6.07), however, scores ≥14 points, which is considered moderate to severe disability, were observed in 39.7% of participants. In the RMQ, the most frequent answers were: “I change position frequently to try and get my back comfortable (84.2%); I avoid heavy jobs around the house because of my back (74.9%); because of my back, I try not to bend or kneel down (73.1%); because of my back, I go upstairs more slowly than usual (62.1%); I walk more slowly than usual because of my back (57.1%); because of my back, I use a handrail to get upstairs (55.5%); I only stand for short periods because of my back (53.3%); because of my back, I lie down to rest more often (50.6%)”.

Table 1 shows sociodemographic characteristics and the association with pain intensity and functional disability in the participants with the presence of LBP. There were 546 (79.6%) female participants, and the mean age of the participants was 67.07 years (SD: 5.98 years). The associations found with sex were for pain (*p* = 0.039) and functional disability (*p* ≤ 0.001); the number of years of schooling was only associated with pain (*p* ≤ 0.001).

The participant’s clinical and behavioral characteristics and their associations with pain intensity and functional disability are shown in Table 2. Around 44.7% had obesity (BMI > 27 kg/m^2^) and were classified as having sedentary (36%) or active (34.7%) lifestyles. When asked whether physical health or emotional problems interfered with normal physical and social activities (family, friends, or groups), 48.3% of the older adults reported that they “in no way” let these factors influence their lives. The perception of health was considered “regular” (46.7%), and the most commonly reported diseases were diabetes mellitus (43.9%) and arterial hypertension (43.9%).

For both pain and functional disability, there were associations with BMI (*p* = 0.048 and *p* < 0.001, respectively), health perception (*p* ≤ 0.001 and *p* < 0.001, respectively), and emotional level (*p* = 0.007 and *p* < 0.001, respectively). Functional disability was associated with self-reported hypertension (*p* = 0.051), diabetes mellitus (*p* = 0.032), dyslipidemia (*p* < 0.001), and physical activity level (*p* < 0.001).

We observed that only the diabetes mellitus (DM) variable was statistically significant, where the PR was 1.24 (*p*-value = 0.0274). In other words, older participants with diabetes mellitus had a 1.24 times higher prevalence of having unbearable pain than participants without the disease (Table 3).

The male sex variable was statistically significant. The PR was 0.66 (*p*-value 0.012), i.e., they were 34% less likely to have a functional disability. The dyslipidemia variable was statistically significant and the PR was 2.14 (*p*-value < 0.001), i.e., they were 2.14 times more likely to have a functional disability. The variable sedentary behavior was statistically significant and the PR was 1.56 (*p*-value < 0.001), i.e., being sedentary was 1.56 times more likely to be associated with a functional disability (Table 4).

## 4. Discussion

The prevalence of LBP was higher in this community of older Amazonia Brazilian adults than in the studies carried out in other Brazilian cities, such as Tabocal in Bahia (18.3 to 23.4%) [28] and São Paulo in São Paulo (25.4%) [29]. A high punctual prevalence of LBP (42.4%) and prevalence for the last 365 days (93.7%) were found.

Regarding spine health, previous studies conducted in Brazil identified that spinal musculoskeletal problems were the second most commonly reported health condition in 2003 and 2008 [30]. In addition, they were also the third most common cause of early retirement due to disability in 2007, creating a high demand for health services amongst older adults [27,30]. Although the prevalence and burden associated with LBP in terms of disability increase with age [31], information about LBP in the older population is minimal, and most studies exclude these individuals.

In this study, the pain score was considered moderate and the functional disability was classified as moderate-to-severe in 39.7% of the participants. This level of functional disability serves as an alert because it shows that it is essential to understand the aspects of the daily living activities that have been reported as difficult to perform because of pain. Dionne et al. [32] carried out a systematic review of all epidemiological studies that examined LBP prevalence by age and identified that people of different ages define disabling pain differently. The authors state that although older adults experience a decrease in non-disabling back pain, their results support the hypothesis that older people less frequently experience or report benign or mild back pain, but they experience a higher prevalence of episodes that are severe or disabling. Furthermore, this is also supported by the findings of Stewart et al. [33], who reported that the frequency of onset of pain that interferes with daily life continues to increase with age.

In fact, in our study, the Amazonas elderly population who lived either in hospital care or in community centers were in the places the interviews were conducted. In other words, our sample differs from the data to others Brazilian cities [11] since the characteristics of the places where data was collected may have contributed to the higher prevalence of low back pain.

Given the social, cultural, and economical fundamental differences between developed and developing countries, it is reasonable to argue that the antecedents and consequences of LBP are not homogeneous. For instance, extreme poverty, infectious epidemic disease, type of occupational activities, family structure, responsibilities, social expectations, geography, and availability and access to health care can have different impacts on the perception and in the reporting of back pain in different contexts [33].

Pain and functional disability were associated with the variables sex, BMI, physical activity level, health perception, and emotional level in our study. These findings are similar to the findings of the study carried out by Hartvigsen et al. [5], which reported that LBP is a complex condition with several contributors to the occurrence of pain; they reported that social, biological, physical, and comorbid factors influence the mechanisms of pain processing and often hamper pain management itself.

Regarding the sex distribution, previous studies showed that women have higher pain prevalence than men [29,34]. This is corroborated by our findings: women were more likely to have LBP compared to men. However, women live longer than men and, thus, are exposed to risk factors for longer; they live with more comorbidities and experience the chronicity of clinical conditions [35]. Murtagh and Hubert [36] described the higher prevalence of disabilities related to health problems in women compared to older men. In a systematic review, the authors suggested that multiple biopsychosocial mechanisms (such as genetics, sex hormones, and pain control) may interact and contribute to the phenomenon [37].

Some studies [38,39] indicate that a high BMI is a risk factor for LBP, corroborating this study’s findings. In line with this evidence, Shiri et al. [40] conducted a systematic review that aimed to investigate the relationship between body weight and LBP, and claimed that overweight and obesity are potentially modifiable risk factors for preventing LBP. In contrast, Stewart et al. [33] and Weiner et al. [41] did not find an association between obesity and disability; however, they evaluated obesity differently compared to our study, where they used waist circumference. In this regard, it is important to highlight the association between these two conditions in this population; although they differ from other studies related to disability due to LBP, these contradictions do not yet provide a conclusive statement.

The prevalence of physical inactivity increases with the aging process [42] and is high amongst the older population worldwide (46.5%) [43]. This is also considered one of the most significant public health problems in modern society [44]. In our study, 36% of the older people were considered sedentary and there was a continuous association between LBP and functional disability. Furthermore, the regression model suggested that the ones who considered themselves sedentary had a 1.65 times higher chance of having a functional disability; this was similar to that found in Canada, wherein sedentary individuals were 33% more likely to have a functional disability than the active ones [45]. Physical activity was an important predictor of functional capacity in the participants with LBP. The more active an older person is, respecting their biological individuality, the better they will live, with improvements not only in musculoskeletal conditions but also in the quality of life and independence [46,47].

Psychological factors influence the disabilities associated with musculoskeletal pain amongst older people [32,39]. The findings of our study regarding the question about LBP and its influence in emotional aspects (“Did physical health or emotional problems interfere with your normal social activities in relation to family, friends, or groups?”) showed an association between pain and functional disability (*p* = 0.007 and *p* < 0.001, respectively). However, the psychological aspects of its influence seemed to vary depending on age and the cultural environment. In addition, psychosocial and emotional problems influence both the chronicity of LBP and pain in general. These conditions are important issues for general practitioners and other professionals dealing with multimorbidity [48]. Moreover, the biopsychosocial consequences of chronic pain emphasize the importance of measuring and investigating its prevalence to plan actions for its control and treatment [29]. To properly address the sociodemographic and psychosocial LBP-related factors within older people, we ought to comprehend the complexity of the pain in its context.

Older people’s self-perception of health (when people evaluate their health status by themselves) was associated with LBP and functional disability. Furthermore, corroborating with these findings, other studies with individuals of various ages showed that LBP’s negative beliefs are associated with a higher level of disability [49,50]. A study conducted only with older adults without specific health conditions found that the higher the degree of dependence, the greater was the chance that the older person perceived their health as bad [51]. It is theorized that for some individuals with LBP, negative beliefs about pain and/or negative illness information lead to a catastrophize response in which the worst possible outcome is imagined. This leads to fear and avoidance of physical activity, which in turn causes resultant distress, reinforcing the original negative appraisal in a deleterious cycle [52].

The present study had some limitations. Participants were recruited using a convenience sample; only the older people who could be at the places where the evaluations took place participated, which may have contributed to a sample selection bias and compromised the generalization of the results. In addition, the older people who were not able to walk outside were not included and those who used a wheelchair were excluded. Furthermore, in this study, people with more serious LBP and more severe impairments were missed. Characteristics such as age, sex, availability, and interest of the participants may have influenced the recruitment and produced discrepancies in the sample’s representativeness; for example, the sample of the present study consisted mostly of women. The proportion of women was higher than that expected from the demographic distribution for the population of older adult women and men in the state of Amazonas. Another limitation was that only the pain intensity scale was used, without using the visual face scale for older adults with lower education levels. On the other hand, we consider our results to be promising for initial public health actions targeting older adults and we encourage more studies because it can be seen as a matter of great importance, as the older population is becoming larger and the costs of treatment are higher than those for preventing the condition.

## 5. Conclusions

In conclusion, the prevalence of LBP was high (punctual prevalence was 42.4% and the prevalence for the last 365 days was 93.7%). Being female, having fewer years of schooling, a higher BMI, engaging in mostly sedentary behavior, having diseases related to the diet and cardiovascular system, or having impaired emotional levels were associated with LBP, even though they perceived themselves in good health. The findings of this study can be used to coordinate efforts from the government, health professionals, and civil society in order to manage and promote the prevention of LBP by considering the elderly population’s needs in the state of Amazonas.

## Figures and Tables

**Table 1:** Sociodemographic characteristics of the studied population (*n* = 700) and the associations found: sociodemographic characteristics, pain intensity, and functional disability in older people with low back pain (*n* = 557).

Variable	Participants *n* = 700 (%)	Pain Median (557)	First Quartile Interval	Third Quartile Interval	*p*-Value	Functional Disability Median (557)	First Quartile Interval	Third Quartile Interval	*p*-Value
Sex									
Female	546 (78)	6	5.00	8.00	0.039 ^1^	12	7.00	17.00	<0.001 ^1^
Male	154 (22)	6	5.00	7.00		9	4.50	12.00	
Age group									
60 to 70 years old	526 (75.1)	6	5.00	8.00	0.651 ^2^	11	6.00	16.00	0.220 ^2^
71 to 80 years old	146 (20.9)	6	5.00	7.00		11	7.00	17.00	
>80 years old	28 (4)	6	5.00	7.00		15	7.00	19.00	
Skin color (self-reported)									
Yellow	27 (3.9)	7	5.50	8.00	0.866 ^2^	11	6.00	16.00	0.790 ^2^
White	196 (28)	6	5.00	8.00		11	7.00	17.00	
Indigenous	18 (2.6)	6	5.00	7.00		12	5.00	15.00	
Brown	387 (55.3)	6	5.00	8.00		11.5	7.00	16.00	
Black	72 (10.3)	6	5.00	8.00		10.5	4.50	16.00	
Marital status									
Married	338 (48.3)	6	5.00	8.00	0.357 ^2^	11	5.50	16.00	0.483 ^2^
Divorced	65 (9.3)	6	4.50	7.00		10	5.00	14.50	
Single	118 (16.9)	6	5.00	8.00		11	7.00	17.00	
Widower	179 (25.6)	6	5.00	7.00		11	8.00	16.00	
Individual income *									
Without income	85 (12.1)	6	5.00	8.00		12.5	5.50	18.00	
Class A	2 (0.3)	7.5	7.00	8.00	0.096 ^2^	17.5	12.00	23.00	0.168 ^2^
Class B	6 (0.9)	6	6.00	7.00		9	0.00	19.00	
Class C	46 (6.6)	5	4.00	7.00		8	4.50	13.50	
Class D	123 (17.6)	6	5.0	7.00		10	7.00	15.00	
Class E	438 (62.6)	6	5.00	8.00		11	6.00	17.00	
Education (years)									
Did not study	3 (0.4)	8	7.00	8.50	<0.001 ^2^	17	10.50	18.50	0.064 ^2^
1 to 4 years	219 (31.3)	6	5.00	8.00		12	7.00	17.00	
5 to 8 years	135 (19.3)	7	5.00	9.00		13	7.00	18.00	
9 to 11 years	76 (10.9)	5	4.00	7.00		10	5.00	15.00	
>11 years	267 (38.1)	6	5.00	7.50		10	5.00	15.00	
Previous occupation **					0.536 ^2^				0.066 ^2^
Armed forces, police, and military Firefighters	2 (0.3)	4	2.00	6.00		6	3.00	9.00	
Science and arts professionals	75 (10.7)	6	5.00	7.00		8	4.00	14.00	
Agricultural, forestry, hunting, and fishing workers	69 (9.9)	6	6.00	7.50		13.5	7.00	17.50	
Industrial goods and service production workers 1	77 (11)	6	5.00	7.50		10	7.50	18.00	
Industrial goods and service production workers 2	29 (4.1)	6	5.00	8.00		11	4.50	16.00	
Administrative service workers	65 (9.3)	6	5.00	6.00		12	5.00	18.00	
Service workers and salespeople in shops and markets	352 (50.3)	6	5.00	7.00		11	7.50	15.00	
Secondary school technicians	29 (4.1)	6	5.00	8.00		12	7.00	16.00	
Current Occupation									
Do not work	634 (90.6)	-		-	-	-	-		-
Work	66 (9.4)	-		-	-	-	-		-

SD: standard deviation; * According to Brazilian Criteria for Economic Classification, 2015; Class A: above BRL 15,760.01; Class B: from BRL 7880.01 to BRL 15.760,00; Class C: from BRL 3152.01 to BRL 7880.00; Class D: from BRL 1576.01 to BRL 3152.00; Class E: up to BRL 1576.00. ** Previous occupation stratified according to the Brazilian Classification of Occupations, 2002. ^1^: Student’s *t*-test or Mann–Whitney; ^2^: ANOVA or Kruskal–Wallis test.

**Table 2:** Clinical and behavioral characteristics of the studied population (*n* = 700) and their associations with pain intensity and functional disability in older adults with low back pain (*n* = 557).

Variable	Participants *n* = 700 (%)	Pain Median (557)	First Quartile Interval	Third Quartile Interval	*p*-Value	Functional Disability Median (557)	First Quartile Interval	Third Quartile Interval	*p*-Value
BMI (kg/m^2^) *									
<22: underweight	77 (11)	6	5.00	8.00	0.048 ^2^	13	7.00	18.00	<0.001 ^2^
22–27: normal weight	310 (44.3)	6	5.00	7.00		10	5.00	15.00	
>27: obesity	313 (44.7)	6	5.00	8.00		12	7.00	17.00	
Physical activity level (IPAQ)									
Active	243 (34.7)	7	5.00	8.00	0.085 ^2^	10	5.00	14.50	<0.001 ^2^
Very active	55 (7.9)	6	4.00	7.00		9	3.50	12.00	
Insufficiently active	150 (21.4)	6	5.00	7.00		10	7.00	16.00	
Sedentary	252 (36)	6	5.00	8.00		14	7.00	18.00	
Smoking									
Nonsmoker	447 (63.9)	6	5.00	8.00	0.245 ^2^	12	7.00	17.00	0.658 ^2^
Ex-smoker	235 (33.6)	6	5.00	8.00		10	6.00	15.00	
Smoker	18 (2.6)	6	6.00	9.00		9	6.00	16.00	
Alcohol consumption									
Do not consume	594 (84.9)	6	5.00	8.00	0.464 ^2^	11	7.00	17.00	0.139 ^2^
Once a month or less	61 (8.7)	6	5.00	7.00		12.5	5.50	15.00	
Twice to four times/month	35 (5.0)	6	4.00	7.50		9	4.00	12.5	
Twice to three times/week	8 (1.1)	5.5	5.00	6.00		7.5	3.00	9.00	
Four or more times/week	2 (0.3)	4.5	4.00	5.00		12.5	8.00	17.00	
Emotional Level **									
No way	338 (48.3)	6	5.00	7.50	0.007 ^2^	8.5	4.00	14.00	<0.001 ^2^
Lightly	172 (24.6)	6	5.00	8.00		14	8.00	18.00	
Moderately	123 (17.6)	6	5.00	7.00		12	8.00	17.00	
Considerable	42 (6)	7	5.00	9.00		15	12.00	18.00	
Extremely	25 (3.6)	8	6.00	10.00		14	11.00	19.00	
Health perception									
Bad	87 (12.4)	8	5.50	10.00	<0.001 ^2^	16	11.00	20.00	<0.001 ^2^
Regular	327 (46.7)	6	5.00	8.00		11	7.00	16.00	
Good	233 (33.3)	6	4.00	7.00		10	4.00	15.00	
Very good	43 (6.1)	6	5.00	6.00		8.5	4.00	11.00	
Excellent	10 (1.4)	4.5	3.00	10.00		4.5	1.00	10.00	
Disease (self-reported) ***									
Diabetes mellitus	160 (28.8)	Absent 6	5.00	8.00	0.077 ^1^	Absent 11	6.00	16.00	0.032 ^1^
		Present 7	5.00	8.00		Present 12	7.00	17.00	
Dyslipidemia	17 (3.1)	Absent 6	5.00	8.00	0.255 ^1^	Absent 11	6.00	16.00	<0.001 ^1^
		Present 6	4.00	6.00		Present 16	14.00	20.00	
Arterial hypertension	244 (43.9)	Absent 6	5.00	8.00	0.521 ^1^	Absent 10	5.00	16.00	0.051 ^1^
		Present 6	5.00	8.00		Present 12	7.00	17.00	
Rheumatoid arthritis	86 (9)	-				-			
Osteoarthritis	99 (11)								
Osteoporosis	22 (2)		-	-	-		-	-	-
Other diseases	62 (6)								
Did not report	169 (17)								

*: Body mass index. **: Did physical health or emotional problems interfere with your normal social activities in relation to family, friends, or groups? ***: Score according to the presence of disease. ^1^: Student’s *t*-test or Mann–Whitney test; ^2^: ANOVA or Kruskal–Wallis test.

**Table 3:** Poisson distribution and the association with unbearable pain.

Variable	Coefficient	SE	PR	95% CI		*p*-Value
				Lower Limit	Higher Limit	
Intercept	−0.5739	0.6291				
Age	−0.0088	0.0083	0.9912	0.9753	10.074	0.2889
Sex (male)	−0.2628	0.1460	0.7689	0.5776	10.236	0.0718
BMI	0.0128	0.0076	10.129	0.9978	10.282	0.0943
DLP	−0.5783	0.4443	0.5609	0.2348	13.398	0.193
DM	0.2163	0.0981	12.415	10.244	15.046	0.0274
Education (5 to 8 years)	0.1408	0.1216	11.512	0.907	1.461	0.247
Education (9 or more years)	−0.1543	0.1141	0.857	0.6853	10.718	0.1761

BMI: body mass index; DLP: dyslipidemia; DM: diabetes mellitus.

**Table 4:** Poisson distribution and association with functional disability.

Variable	Coefficient	SE	PR	95% CI		*p*-Value
				Lower Limit	Higher Limit	
Intercept	−19.871	0.6224				0.0014
Age	0.0054	0.0082	10.054	0.9895	10.216	0.5064
Sex (male)	−0.4032	0.1607	0.6682	0.4877	0.9156	0.0121
BMI	0.0146	0.0080	10.147	0.9989	10.308	0.0684
Arterial hypertension	0.1067	0.1019	11.126	0.9111	13.585	0.2952
DLP	0.7629	0.1537	21.445	15.868	28.982	<0.0001
DM	0.1357	0.1030	11.453	0.9359	14.014	0.1877
Education (5 to 8 years)	0.2146	0.1341	12.394	0.9529	16.119	0.1095
Education (9 or more years)	−0.0093	0.1238	0.9907	0.7773	12.626	0.9399
Physical activity level (sedentary)	0.4471	0.1039	15.638	12.757	19.171	<0.0001

BMI: body mass index; DLP: dyslipidemia; DM: diabetes mellitus.

## Data Availability

The data presented in this study are available on request from the corresponding author. The data are not publicly available due to personal participation information.
